# *catena*-Poly[[(2-amino­pyrimidine-κ*N* ^1^)(thio­cyanato-κ*S*)mercury(II)]-μ-thio­cyanato-κ^2^ *S*:*N*]

**DOI:** 10.1107/S1600536812016790

**Published:** 2012-04-21

**Authors:** Fatemeh Hoseinzadeh, Sadif A. Shirvan, Sara Haydari Dezfuli

**Affiliations:** a Technical and Vocational University, Ozgoli Square, Lavizan, Tehran, Iran; b Department of Chemistry, Islamic Azad University, Omidieh Branch, Omidieh, Iran

## Abstract

In the title coordination polymer, [Hg(NCS)_2_(C_4_H_5_N_3_)], the Hg^II^ atom is four-coordinated by one aromatic N atom from a 2-amino­pyrimidine ligand, one S atom from a terminal thio­cyanate ligand, and one S atom and one N atom from a bridging thio­cyanate ligand. The crystal structure features polymeric chains running along the *b* axis which are stabilized by N—H⋯N hydrogen bonds.

## Related literature

For related structures with amino­pyridine as a ligand, see: Albada *et al.* (2002 ▶); Castillo *et al.* (2011 ▶); Cheng *et al.* (2009 ▶); Cui *et al.* (2011 ▶); Gao & Ng (2010 ▶); Lee *et al.* (2003 ▶); Li *et al.* (2006 ▶); Lin & Zeng (2007 ▶); Masaki *et al.* (2002 ▶); Qu *et al.* (2008 ▶); Zhu *et al.* (2002 ▶, 2003 ▶).
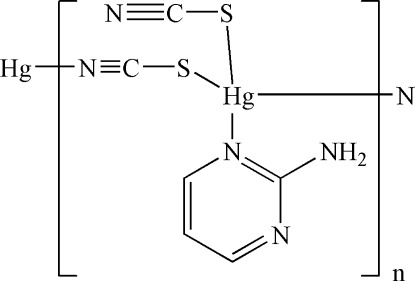


## Experimental

### 

#### Crystal data

[Hg(NCS)_2_(C_4_H_5_N_3_)]
*M* *_r_* = 411.88
Monoclinic, 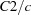

*a* = 25.819 (2) Å
*b* = 6.0060 (4) Å
*c* = 20.1176 (15) Å
β = 136.222 (4)°
*V* = 2158.4 (3) Å^3^
*Z* = 8
Mo *K*α radiation
μ = 14.62 mm^−1^
*T* = 298 K
0.25 × 0.22 × 0.11 mm

#### Data collection

Bruker APEXII CCD area-detector diffractometer
Absorption correction: numerical (*SADABS*; Bruker, 2001 ▶) *T*_min_ = 0.039, *T*_max_ = 0.222
7675 measured reflections
2131 independent reflections
1671 reflections with *I* > 2σ(*I*)
*R*_int_ = 0.078

#### Refinement

*R*[*F* ^2^ > 2σ(*F* ^2^)] = 0.049
*wR*(*F* ^2^) = 0.088
*S* = 1.10
2131 reflections
127 parameters
H-atom parameters constrained
Δρ_max_ = 0.72 e Å^−3^
Δρ_min_ = −1.23 e Å^−3^

### 

Data collection: *APEX2* (Bruker, 2007 ▶); cell refinement: *SAINT* (Bruker, 2007 ▶); data reduction: *SAINT*; program(s) used to solve structure: *SHELXS97* (Sheldrick, 2008 ▶); program(s) used to refine structure: *SHELXL97* (Sheldrick, 2008 ▶); molecular graphics: *ORTEP-3 for Windows* (Farrugia, 1997 ▶); software used to prepare material for publication: *WinGX* (Farrugia, 1999 ▶).

## Supplementary Material

Crystal structure: contains datablock(s) I, global. DOI: 10.1107/S1600536812016790/bt5873sup1.cif

Structure factors: contains datablock(s) I. DOI: 10.1107/S1600536812016790/bt5873Isup2.hkl

Additional supplementary materials:  crystallographic information; 3D view; checkCIF report

## Figures and Tables

**Table 1:** Hydrogen-bond geometry (Å, °)

*D*—H⋯*A*	*D*—H	H⋯*A*	*D*⋯*A*	*D*—H⋯*A*
N1—H1*A*⋯N5^i^	0.86	2.21	3.052 (14)	167
N1—H1*B*⋯N3^ii^	0.86	2.15	3.004 (13)	176

Symmetry codes: (i) 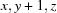; (ii) 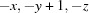.

**Fig. 1.**
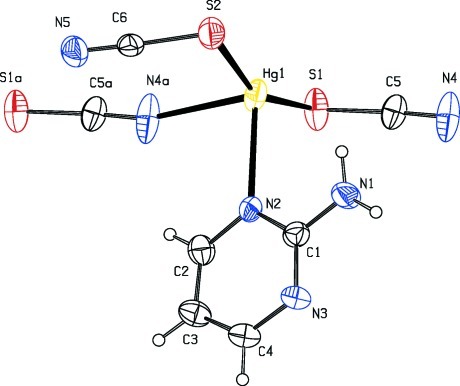
The molecular structure of the title molecule, with the atom-numbering scheme. Displacement ellipsoids are drawn at the 30% probability level. [Symmetry codes: (a) x,-1 + y,z].

**Fig. 2.**
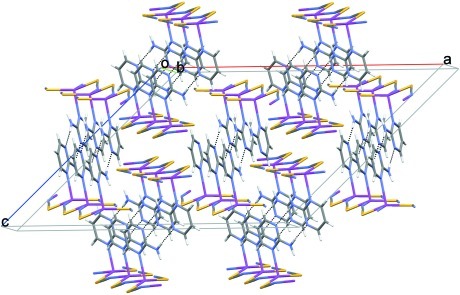
Unit-cell packing diagram for title molecule. Hydrogen bonds are shown as dashed lines.

## supplementary crystallographic information

### Comment

Numerous complexes with 2-aminopyrimidine as a ligand have been prepared, such as that of cobalt (Li *et al.*, 2006), manganese (Lee *et al.*, 2003), nickel (Masaki *et al.*, 2002), zinc (Gao *et al.*, 2010; Qu *et al.*, 2008; Lin & Zeng, 2007), cadmium (Castillo *et al.*, 2011; Cheng *et al.*, 2009), silver (Zhu *et al.*, 2003; Cui *et al.*, 2011) and copper (Zhu *et al.*, 2002; Albada *et al.*, 2002). Here, we report the synthesis and structure of the title compound.

In the title coordination polymer, (Fig. 1), the Hg^II^ atom is four-coordinated in a butterfly configuration by one N atom from one 2-aminopyrimidine, one S atom from one terminal SCN ligand, one S atom from one bridging SCN and one N atom from one bridging SCN ligand.

In the crystal structure, intermolecular N—H···N hydrogen bonds (Table 2, Fig. 2) stabilize the structure.

### Experimental

A solution of 2-aminopyrimidine (0.19 g, 2.0 mmol) in methanol (20 ml) was added to a solution of Hg(SCN)_2_ (0.43 g, 1.0 mmol) in methanol (20 ml) and the resulting colorless solution was stirred for 20 min at 313 K. This solution was left to evaporate slowly at room temperature. After one week, colourless prismatic crystals of the title compound were isolated (yield; 0.33 g, 72.8%).

### Refinement

All H atoms were positioned geometrically, with C—H = 0.93 Å and N—H = 0.86 Å and constrained to ride on their parent atoms with *U*_iso_(H)=1.2*U*_eq_(C,N).

### Crystal data

**Table:**

[Hg(NCS)_2_(C_4_H_5_N_3_)]	*F*(000) = 1504
*M**_r_* = 411.88	*D*_x_ = 2.535 Mg m^−^^3^
Monoclinic, *C*2/*c*	Mo *K*α radiation, λ = 0.71073 Å
Hall symbol: -C 2yc	Cell parameters from 7675 reflections
*a* = 25.819 (2) Å	θ = 2.9–26.0°
*b* = 6.0060 (4) Å	µ = 14.62 mm^−^^1^
*c* = 20.1176 (15) Å	*T* = 298 K
β = 136.222 (4)°	Prism, colorless
*V* = 2158.4 (3) Å^3^	0.25 × 0.22 × 0.11 mm
*Z* = 8	

### Data collection

**Table:**

Bruker APEXII CCD area-detector diffractometer	2131 independent reflections
Radiation source: fine-focus sealed tube	1671 reflections with *I* > 2σ(*I*)
Graphite monochromator	*R*_int_ = 0.078
ω scans	θ_max_ = 26.0°, θ_min_ = 2.9°
Absorption correction: numerical (*SADABS*; Bruker, 2001)	*h* = −31→27
*T*_min_ = 0.039, *T*_max_ = 0.222	*k* = −7→7
7675 measured reflections	*l* = −23→24

### Refinement

**Table:**

Refinement on *F*^2^	Primary atom site location: structure-invariant direct methods
Least-squares matrix: full	Secondary atom site location: difference Fourier map
*R*[*F*^2^ > 2σ(*F*^2^)] = 0.049	Hydrogen site location: inferred from neighbouring sites
*wR*(*F*^2^) = 0.088	H-atom parameters constrained
*S* = 1.10	*w* = 1/[σ^2^(*F*_o_^2^) + (0.0349*P*)^2^] where *P* = (*F*_o_^2^ + 2*F*_c_^2^)/3
2131 reflections	(Δ/σ)_max_ = 0.008
127 parameters	Δρ_max_ = 0.72 e Å^−^^3^
0 restraints	Δρ_min_ = −1.23 e Å^−^^3^

### Special details

**Table:**

Geometry. All e.s.d.'s (except the e.s.d. in the dihedral angle between two l.s. planes) are estimated using the full covariance matrix. The cell e.s.d.'s are taken into account individually in the estimation of e.s.d.'s in distances, angles and torsion angles; correlations between e.s.d.'s in cell parameters are only used when they are defined by crystal symmetry. An approximate (isotropic) treatment of cell e.s.d.'s is used for estimating e.s.d.'s involving l.s. planes.
Refinement. Refinement of *F*^2^ against ALL reflections. The weighted *R*-factor *wR* and goodness of fit *S* are based on *F*^2^, conventional *R*-factors *R* are based on *F*, with *F* set to zero for negative *F*^2^. The threshold expression of *F*^2^ > σ(*F*^2^) is used only for calculating *R*-factors(gt) *etc*. and is not relevant to the choice of reflections for refinement. *R*-factors based on *F*^2^ are statistically about twice as large as those based on *F*, and *R*- factors based on ALL data will be even larger.

### Fractional atomic coordinates and isotropic or equivalent isotropic displacement parameters (Å^2^)

**Table:**

	*x*	*y*	*z*	*U*_iso_*/*U*_eq_	
Hg1	0.16617 (2)	0.10942 (6)	0.33894 (3)	0.05561 (16)	
S1	0.1305 (2)	0.3052 (4)	0.4049 (2)	0.0707 (8)	
S2	0.25302 (16)	−0.0298 (4)	0.3378 (2)	0.0641 (7)	
C1	0.0285 (5)	0.2478 (15)	0.1021 (6)	0.049 (2)	
C2	0.0007 (6)	−0.0719 (16)	0.1318 (7)	0.055 (2)	
H2	0.0126	−0.1780	0.1749	0.067*	
C3	−0.0648 (5)	−0.0935 (19)	0.0381 (7)	0.058 (3)	
H3	−0.0961	−0.2152	0.0153	0.069*	
C4	−0.0821 (5)	0.0738 (17)	−0.0211 (7)	0.054 (3)	
H4	−0.1278	0.0675	−0.0855	0.065*	
C5	0.1323 (6)	0.5655 (15)	0.3790 (8)	0.055 (3)	
C6	0.2379 (5)	−0.3009 (18)	0.3346 (6)	0.047 (2)	
N1	0.0752 (5)	0.4154 (14)	0.1322 (6)	0.073 (3)	
H1A	0.1176	0.4234	0.1916	0.088*	
H1B	0.0629	0.5155	0.0923	0.088*	
N2	0.0491 (4)	0.0940 (11)	0.1659 (5)	0.0437 (16)	
N3	−0.0368 (4)	0.2472 (13)	0.0082 (5)	0.051 (2)	
N4	0.1294 (7)	0.7461 (14)	0.3608 (9)	0.090 (4)	
N5	0.2301 (5)	−0.4879 (17)	0.3329 (7)	0.070 (3)	

### Atomic displacement parameters (Å^2^)

**Table:**

	*U*^11^	*U*^22^	*U*^33^	*U*^12^	*U*^13^	*U*^23^
Hg1	0.0759 (3)	0.03563 (19)	0.0604 (2)	0.0129 (2)	0.0510 (2)	0.0059 (2)
S1	0.123 (3)	0.0400 (12)	0.097 (2)	−0.0033 (15)	0.096 (2)	−0.0014 (14)
S2	0.0652 (17)	0.0482 (13)	0.0875 (19)	−0.0009 (13)	0.0581 (16)	−0.0002 (13)
C1	0.047 (5)	0.049 (5)	0.041 (5)	0.004 (4)	0.029 (5)	−0.001 (4)
C2	0.062 (6)	0.050 (6)	0.063 (6)	−0.005 (5)	0.048 (6)	0.002 (5)
C3	0.047 (5)	0.073 (7)	0.058 (6)	−0.013 (5)	0.039 (5)	−0.005 (6)
C4	0.039 (5)	0.072 (7)	0.045 (5)	−0.006 (5)	0.028 (4)	−0.004 (5)
C5	0.080 (7)	0.045 (6)	0.074 (7)	0.010 (5)	0.067 (6)	0.013 (5)
C6	0.033 (5)	0.050 (5)	0.049 (5)	0.001 (4)	0.026 (5)	−0.005 (4)
N1	0.062 (5)	0.058 (5)	0.043 (4)	−0.017 (4)	0.018 (4)	0.007 (4)
N2	0.047 (4)	0.036 (4)	0.042 (4)	0.004 (3)	0.030 (3)	0.006 (3)
N3	0.037 (4)	0.062 (5)	0.042 (4)	−0.007 (4)	0.024 (4)	−0.005 (4)
N4	0.163 (11)	0.036 (5)	0.164 (11)	0.016 (6)	0.148 (10)	0.014 (6)
N5	0.063 (6)	0.051 (5)	0.089 (7)	−0.004 (5)	0.053 (6)	−0.016 (5)

### Geometric parameters (Å, º)

**Table:**

Hg1—S1	2.399 (5)	N3—C4	1.343 (16)
Hg1—S2	2.409 (5)	N4—C5	1.130 (13)
Hg1—N2	2.464 (7)	N5—C6	1.137 (15)
Hg1—N4^i^	2.542 (13)	N1—H1B	0.8600
S1—C5	1.659 (11)	N1—H1A	0.8600
S2—C6	1.665 (11)	C2—C3	1.352 (15)
N1—C1	1.334 (16)	C3—C4	1.364 (16)
N2—C1	1.341 (12)	C2—H2	0.9300
N2—C2	1.337 (16)	C3—H3	0.9300
N3—C1	1.346 (12)	C4—H4	0.9300
			
Hg1···N5^ii^	2.980 (13)	C2···S1^iii^	3.679 (16)
Hg1···H1A	2.9200	C3···S1^iii^	3.569 (15)
S1···N4^i^	3.468 (10)	C4···S1^iii^	3.630 (15)
S1···C2^iii^	3.679 (16)	C4···N5^vii^	3.44 (2)
S1···C3^iii^	3.569 (15)	C4···C1^vi^	3.400 (19)
S1···C4^iii^	3.630 (15)	C5···C6^ii^	3.52 (2)
S2···N5^ii^	3.297 (11)	C5···N5^ii^	3.27 (2)
S2···H3^iv^	3.1900	C6···C5^i^	3.52 (2)
N1···N5^ii^	3.052 (14)	C1···H1B^v^	3.0900
N1···N3^v^	3.004 (13)	C4···H1B^v^	3.0700
N3···N1^v^	3.004 (13)	C6···H1A^i^	2.7900
N4···C6^ii^	3.22 (3)	C6···H3^iv^	3.0200
N4···S1^ii^	3.468 (10)	C6···H4^iv^	3.0200
N4···C2^ii^	3.370 (16)	H1A···Hg1	2.9200
N5···N1^i^	3.052 (14)	H1A···N5^ii^	2.2100
N5···C4^iv^	3.44 (2)	H1A···C6^ii^	2.7900
N5···Hg1^i^	2.980 (13)	H1B···N3^v^	2.1500
N5···S2^i^	3.297 (11)	H1B···C1^v^	3.0900
N5···C5^i^	3.27 (2)	H1B···C4^v^	3.0700
N3···H1B^v^	2.1500	H2···N4^i^	2.6500
N4···H2^ii^	2.6500	H3···S2^vii^	3.1900
N5···H1A^i^	2.2100	H3···C6^vii^	3.0200
N5···H4^iv^	2.7700	H4···N5^vii^	2.7700
C1···C4^vi^	3.400 (19)	H4···C6^vii^	3.0200
			
S1—Hg1—S2	155.11 (12)	C1—N1—H1B	120.00
S1—Hg1—N2	103.3 (3)	N1—C1—N2	118.8 (8)
S1—Hg1—N4^i^	89.1 (4)	N1—C1—N3	116.4 (9)
S2—Hg1—N2	100.0 (3)	N2—C1—N3	124.8 (10)
S2—Hg1—N4^i^	99.8 (5)	N2—C2—C3	123.5 (10)
N2—Hg1—N4^i^	89.0 (4)	C2—C3—C4	116.0 (11)
Hg1—S1—C5	100.3 (6)	N3—C4—C3	123.8 (10)
Hg1—S2—C6	98.4 (6)	S1—C5—N4	175 (2)
Hg1—N2—C1	124.1 (7)	S2—C6—N5	177.0 (15)
Hg1—N2—C2	119.5 (6)	N2—C2—H2	118.00
C1—N2—C2	116.4 (8)	C3—C2—H2	118.00
C1—N3—C4	115.4 (9)	C2—C3—H3	122.00
Hg1^ii^—N4—C5	159.2 (18)	C4—C3—H3	122.00
H1A—N1—H1B	120.00	N3—C4—H4	118.00
C1—N1—H1A	120.00	C3—C4—H4	118.00
			
S2—Hg1—S1—C5	79.5 (5)	S2—Hg1—N4^i^—C5^i^	18 (3)
N2—Hg1—S1—C5	−80.0 (5)	N2—Hg1—N4^i^—C5^i^	118 (3)
N4^i^—Hg1—S1—C5	−168.8 (6)	Hg1—N2—C1—N1	3.2 (18)
S1—Hg1—S2—C6	118.0 (4)	C2—N2—C1—N1	−179.2 (13)
N2—Hg1—S2—C6	−82.3 (4)	Hg1—N2—C1—N3	−176.8 (10)
N4^i^—Hg1—S2—C6	8.4 (4)	C2—N2—C1—N3	1 (2)
S1—Hg1—N2—C1	91.2 (11)	C1—N2—C2—C3	2 (2)
S1—Hg1—N2—C2	−86.3 (10)	Hg1—N2—C2—C3	180.0 (12)
S2—Hg1—N2—C1	−80.2 (10)	C4—N3—C1—N2	−2 (2)
S2—Hg1—N2—C2	102.3 (11)	C4—N3—C1—N1	178.3 (13)
N4^i^—Hg1—N2—C1	−179.9 (11)	C1—N3—C4—C3	0 (2)
N4^i^—Hg1—N2—C2	2.5 (11)	N2—C2—C3—C4	−4 (2)
S1—Hg1—N4^i^—C5^i^	−139 (3)	C2—C3—C4—N3	3 (2)

Symmetry codes: (i) *x*, *y*−1, *z*; (ii) *x*, *y*+1, *z*; (iii) −*x*, *y*, −*z*+1/2; (iv) *x*+1/2, −*y*−1/2, *z*+1/2; (v) −*x*, −*y*+1, −*z*; (vi) −*x*, −*y*, −*z*; (vii) *x*−1/2, −*y*−1/2, *z*−1/2.

### Hydrogen-bond geometry (Å, º)

**Table:**

*D*—H···*A*	*D*—H	H···*A*	*D*···*A*	*D*—H···*A*
N1—H1*A*···N5^ii^	0.86	2.21	3.052 (14)	167
N1—H1*B*···N3^v^	0.86	2.15	3.004 (13)	176

Symmetry codes: (ii) *x*, *y*+1, *z*; (v) −*x*, −*y*+1, −*z*.
